# “She Was Feeling Overwhelmed at Home Caring for Her Children”: Expectations of “Intensive Motherhood” as a Risk Factor for Young Women's Suicide

**DOI:** 10.1177/10778012241265365

**Published:** 2024-07-23

**Authors:** Harriet Townsend

**Affiliations:** 1 7788 Macquarie University, Sydney, NSW, Australia

**Keywords:** suicide, motherhood, suicidal femininity, gender

## Abstract

Suicide is a leading cause of death among young women and perinatal mothers. This paper explores how expectations of motherhood played a role in young women's deaths by suicide. I question the notion that motherhood is a “protective” factor against suicide. Using the concept of “intensive motherhood,” I interrogate how expectations of mothers became fatal. Through analysis of 31 young Australian mothers who died by suicide, three key themes are explored, centered upon the theme of “failing motherhood”: mothering without a father, mothering with mental illness, and the loss of care of children.

## Introduction

Suicide is a leading cause of death among both young women (aged 15–24 years) and women in the perinatal period in Australia, which includes pregnancy and the 12 months following birth (ABS, 2023a; AIHW, 2023a, 2023b; Chin et al., 2022; Meurk et al., 2021). Motherhood is generally considered protective against suicide, and suicide among mothers is considered taboo (Al-Halabí et al., 2021). Research into maternal suicide focuses on the perinatal period (see Chin et al., 2022; Modini et al., 2021), likely due to the high risk of psychiatric disorders during this time (Appleby, 1991; Khalifeh et al., 2016; Modini et al., 2021; Qin & Mortensen, 2003). This is despite burgeoning evidence that mothers of non-adult children are more likely to suicide *after* the perinatal period (Lysell et al., 2018; Sörberg Wallin et al., 2022). Some research has endeavored to understand the risks associated with suicidality (which includes suicidal thoughts, plans, nonfatal^1^ suicides, and deaths by suicide) among perinatal mothers. However, due to the use of quantitative methodologies, this research tends to repeatedly identify the same risks such as mental illness, family violence, lack of social support, history of abuse, unplanned pregnancy, pregnancy loss, and other adversities (Biaggi et al., 2016; Chin et al., 2022; Hjelmland & Knizek, 2016; Meurk et al., 2021). Furthermore, these studies frame suicide as a mental health problem rather than a social one (Marsh, 2016; White et al., 2016) and so provide a limited understanding of why people suicide. Research into suicidality among mothers is no different.

There is little research on suicide by mothers following the perinatal period. This gives the impression that issues associated with identified risk factors for suicidality disappear upon the child's first birthday. Once a mother's child turns one, intellectual interest in her suicidality wanes, and she generally becomes like most women studied in the field of suicidology: an incidental comparator to men who died by suicide, used as confirmation for the prioritization of suicide prevention efforts toward men (Mallon, 2018; Mallon et al., 2016).

Furthermore, there is limited national-level research into deaths by suicide among young people in Australia (Hill et al., 2021), particularly young women (Robinson et al., 2016; Rugkhla, 2011). This is despite national statistics indicating a steady increase in suicide rates among young women (ABS, 2023b; Stefanac et al., 2019). Suicidality has remained high among young women in Australia, indicated by their highest rates of hospitalizations for suicidal and non-suicidal self-harm, and ambulance attendances for suicidal ideation and non-fatal suicides (AIHW, 2023c; 2023d).

Though national statistics show that suicidality is high among women and that there is an increasing trend in deaths by suicide among young women, there is little interest in understanding the women who suicide or the role of femininity in their suicides (Mallon, 2018; Mallon et al., 2016). However, there are numerous studies which aim to understand the role of masculinity among men who suicide (see Scotti Requena et al., 2024). This is in part due to the over-reliance in suicidology on large-scale quantitative datasets (Hjelmeland, 2016; Hjelmeland & Knizek, 2016) which demonstrate the high prevalence of *deaths* by suicide among men. The high number of male deaths by suicide has been used to justify prioritizing male suicide prevention within policy and research (for an example see Suicide Prevention Australia, 2023. See also Jaworski, 2010; Jaworski, 2016; McKay et al., 2014). Qualitative research is often used as a means of attempting to understand the role of gender among people who die by suicide because it allows for a better understanding of how cultural and social structures, including gender, may have affected individuals’ suicidality (see Bennett et al., 2023, Scourfield, 2005; Scourfield et al., 2012; Shiner et al., 2009). However, these studies are also dominated by understandings of suicide through the lens of masculinities.

This paper responds to the lacuna in research about young women inside and outside the perinatal period. It examines expectations of “intensive” motherhood and adds to debates regarding motherhood as protective against suicide. The implications are two-fold: to broaden understandings of the conditions under which young women die by suicide and the role of norms of motherhood in these deaths, and to provide some insights into the lives of young mothers who die by suicide outside of the perinatal period. This paper focuses on the experience of young women who are mothers because these women are considered antithetical to the norms of intensive motherhood in many contexts, including Australia. Pregnancy and motherhood among teens, for example, remain sources stigma in many places, clashing with life course expectations of appropriate teenage behavior and good mothering (Ellis-Sloane 2014, Sharpe 2015, Yardley 2008). Some women feel pressured to attain personal goals such as travel, career, or education before childbirth to ensure they can emotionally invest in and financially provide for their children, meaning they need to have children after their mid-20s (Ngu et al., 2015). As a result, young mothers may be viewed as immature, irresponsible, and unprepared for parenthood (Francombe-Webb & Silk, 2016; Mangeli et al., 2017). These women therefore present an illustrative case study of the role of normative expectations around motherhood as well as understanding of suicide among mothers and young women more generally.

The article proceeds by examining current debates around motherhood as protective against suicide. It then provides an overview of expectations of motherhood with a specific look at Hays’ (1996) theory of “intensive motherhood” which examines how normative expectations of motherhood are contradictory to other expectations of women generally, for example, paid employment. The following section presents an overview of the methodology used to analyze and report the results. The paper then analyzes 31 cases of young mothers who died by suicide, whose case files were then analyzed thematically. It explores three key themes related to different normative expectations of intensive motherhood that help us understand the significance of intensive motherhood as a factor in young women's suicides. The implications of these findings are discussed in the final sections of the article.

### Motherhood as a Protective Factor Against or Risk Factor for Suicide?

Despite little evidence to confirm it, motherhood is generally viewed as protective against suicide among mothers (Al-Halabí et al., 2021; Appleby, 1991; Qin & Mortensen, 2003). As noted above, research which does look at suicidality among mothers is restricted to perinatal mothers (see Chin et al., 2022; Modini et al., 2021). Expectations of gender permeate reasons provided for why motherhood or parenthood could be protective against suicide, for example, the postulation that babies can bring a sense of self-worth and a feeling of being needed (Appleby, 1991; Qin & Mortensen, 2003). Recently, the protective power of motherhood among perinatal women who died by suicide has been called into question and continues to be debated (Chin et al., 2022). However, these quantitative studies continue to suggest and assume reasons why mothers become suicidal instead of drawing evidence from qualitative reviews of the experiences of women who died by suicide, for example, from coronial case-files, and/or discussion with women who have experienced nonfatal suicide and suicidality.

In the few studies which have examined suicidality by talking to mothers about their experience using qualitative methods, it appears that motherhood, at least for these women, was a contributing factor to their decision to suicide and perhaps, in some cases, should be considered a risk factor for suicide. Reid et al. (2022) found a number of factors that led perinatal women to feel like failures and subsequently self-identify as “bad mothers.” Beliefs of being a bad mother exacerbated feelings of shame and guilt that added to the relentlessness of motherhood, resulting in some women becoming suicidal (Reid et al., 2022). These sentiments of shame were echoed by mothers who had experienced nonfatal suicide in a study on the suicidality of perinatal women conducted by Biggs et al. (2023). Women in that study experienced shame so deeply that suicide became a logical choice and a means of dealing with the shame. These studies indicate that motherhood, at least for some women, may be a risk factor for rather than protective against suicide. These studies attempt to help us understand why women become suicidal by talking to them about their experiences, rather than assuming or postulating why they might have become suicidal. However, they too are restricted to solely focusing on understanding women during the perinatal period and do not analyze the results using theories of gender. As a result, we are unable to see how social structures and forces may have affected these women (Jaworski, 2010; Jaworski, 2016).

As they stand, suicide studies among mothers overemphasize understandings of suicide promoted within large quantitative studies and do little to understand more nuanced experience. This gap in understanding can be addressed by using qualitative methods to provide context to and document variations of experience among mothers who died by suicide (Hjelmeland & Knizek, 2010, 2016).

### Gendered Expectations of “Intensive” Motherhood

Understandings of children, motherhood, and parenthood are socially constructed and deeply gendered (Hays, 1996). They are performed (Butler, 1993; Risman, 2004) and are also a frame of reference for how to think, feel, and behave (Ridgeway, 2009). Gender is hierarchical (Schippers, 2007). Women who transgress expectations of hegemonic, idealized femininity, by, for example, being overweight, non-white, poor, single, and without the desire for children, are stigmatized while those who can meet these hegemonic expectations are rewarded (Schippers, 2007). Gender is also iterative: expectations, like those of motherhood, can be understood as though they are “natural” rather than socially constructed (Butler, 1993).

If we understand motherhood as culturally constructed and gendered, we can see how people are led to believe that pregnancy and motherhood are “natural” parts of womanhood, and therefore, a woman will know exactly how to “be” a mother once she has her baby and, from then, only joy will ensue (Chrisler, 2013). These sentiments are enduring cultural artifacts from the patriarchal institution of “motherhood” which aims to continue the oppression and submission of women to men (O’Reilly, 2020:57). If these expectations do not manifest, a mother may feel guilt, shame, and inadequacy (Henderson et al., 2016).

A “good” mother is described as “patient, kind, nurturing, receptive, gentle, and soft-spoken,” and therefore, a “bad” mother is characterized as “annoyed, angry, impatient, loud, frustrated, bored, turned inward, or otherwise unapproachable” (Chrisler, 2013:118). A “good” mother is not only the primary caregiver but also “child-centered,” meaning she provides physical, emotional, and financial support in abundance (Huisman & Joy, 2014; Takseva, 2014). In contrast, a “bad” mother is overly focused on her career or herself and is financially unable to provide—even though commitment to work is often *how* she can financially provide for herself and children. To achieve both financial security as well as sufficient time for her children, she needs to therefore be partnered with someone who works and contributes financially, ideally a man. These behaviors are reflected in Hays’ (1996) concept of “intensive mothering,” a concept used throughout this paper to connote notions of what is expected of “good” mothers.

Hays (1996) outlines how expectations of motherhood, in particular intensive motherhood, are contradictory and constructed in a way such that no mother or person can fulfill. The intensive mother should, most importantly, put her child's needs above all else—in particular her own (Hays, 1996). An essential part of intensive mothering is, therefore, that a woman ought to maintain a happy, loving, and monogamous relationship with her male partner, as this is what is considered “best” for the children (Hays, 1996). These expectations fetishize ideals associated with the traditional nuclear family, where mothers stay home and look after the children, take care of the house, and tend to their husband when he returns from work, ideals proportions of women in Australia and elsewhere still aspire to (Bugden et al., 2021).

To never have or to lose these signifiers of a happy partnership and family can have negative effects on women. Single mothers are noted to have generally higher levels of stress, anxiety, and depression than partnered mothers (Biaggi et al., 2016), except, perhaps unsurprisingly, when the relationship is unsupportive (Bilszta et al., 2008). Some single mothers have voiced feelings of shame for not living up to the ideal of parenthood by providing loving, safe, and forever homes with a mother and a father (Johnson, 2014; Morris & Munt, 2019). Furthermore, these expectations can have negative consequences on people and parents who are not heterosexual or cis-gendered (Charter et al., 2018; Ross et al., 2008).

Intensive motherhood is therefore heteronormative, racist, ableist, and classed and can create stigma and shame for those who do not or cannot conform. In particular, mothers who are non-white, young, single, and poor are constructed as figures of contempt and disgust (Tyler, 2008) through the stereotype of “the welfare queen” (Hancock, 2003). “Welfare Queens” are assumed to be dependent upon the state rather than themselves for their families’ survival (Hancock, 2003). These connotations are bound up with notions of neo-liberal individualism which focus on individual responsibility over one's “choices” and render invisible individual, cultural, and institutional effects on women's lives, such as gender, sexism, and racism (Bugden et al., 2021). Though Hays (1996) noted the contradictions and difficulties many mothers face—these issues are experienced differently by women of varying class backgrounds, ethnicities, cultural backgrounds, and abilities (Gross et al., 2014). Variations in experiences of motherhood and the expectations of it, in turn, affect how women access, use, receive, and retain informal and formal systems of care and support, the latter of which have been found to be paternalistic in their expectations of “good” mothers (Gross et al., 2014). This can have devastating effects on mothers who need help.

According to Hays (1996), an intensive mother is the primary caregiver, expertly guided and physically, emotionally, and financially absorbed by her children. Implicit in this is that her children's wellbeing is her primary concern and responsibility. Of course, not all mothers are able to achieve these expectations, and are deemed “risky.” Fears over child safety have become a central part of modern intensive motherhood (Brown, 2014). The implication of being a risky mother can remain even when the risk posed is not from women themselves, but from violent partners and/or family members, because of the assumption that mothers are responsible for preventing violence (Bordo, 1993). Expectations of intensive motherhood therefore put an unequal burden of responsibility upon women in situations which are not conducive to intensive motherhood. Sometimes, if they are unable to meet these expectations, the state might intervene (Gross et al., 2014). Again, we see here the neo-liberal notion of individual choice rendering invisible other factors which affect women's lives. These factors include that, in Australia for example, there are chronic shortages of emergency accommodation and refuges for women and children – (AIHW, 2022) and there is an assumption that women are responsible for ending men's violence.

Hays (1996) explains intensive motherhood is contradictory to other demands on a woman's time. For example, a mother should look after her own mental wellbeing so that she can continue to mother intensively (Brown, 2014). Yet being a good patient can be incongruous with the realities of being a good mother, including a lack of time for self-care due to the demands of mothering intensively. Hays’ (1996) theory of intensive motherhood does not discuss mental illness and postnatal depression explicitly; however, the connotations of mothers being warm towards, well bonded with, and emotionally absorbed in children are aspects of intensive motherhood (Hays, 1996:46–7). Intensive motherhood has since been applied to the mental health of mothers (see, for example, Brown, 2014; Rizzo et al., 2012). Intensive motherhood ideals can be discordant with the experiences of mothers suffering from postnatal depression and other mental illnesses and can negatively affect mental health (Rizzo et al., 2012).

Expectations of medical help-seeking, particularly for mental illness, contradict expectations of intensive motherhood because they require the woman to put her needs first even though, the goal is to ensure that she can continue to devote herself to her children (Brown, 2014). Deviation from these expectations is understood to be a failing of the individual, and responsibility for not living up to these standards is presumed to be entirely the mothers’ failing, rather than a failing of the social and political structures that do not support her to parent, work, and have leisure as she sees fit (Brown, 2014; Bugden et al., 2021).

Intensive motherhood remains an important cultural theory to explain how expectations of gender affect women and mothers. The application of intensive motherhood helps us uncover and understand the effects of social and cultural forces on women and mothers. The present study aims to broaden understanding of young mothers who died by suicide by applying intensive motherhood to gain a feminist and gendered perspective on young women's suicides. I ask the question: how did the notion of motherhood being protective against suicide affect young mothers who died by suicide? Because the notion of motherhood is bound up with expectations of gender, the question underpinning this is: how did expectations of motherhood affect young suicidal mothers? The overall aim is to understand suicidal femininities generally and how gendered expectations can have fatal consequences among young mothers.

## Sample and Methodology

Cases were identified from the National Coronial Information System (NCIS), a database of all coronial files in Australia dating back to 2000 (except Queensland which began in 2001) and New Zealand since 2007 (NCIS 2018).^2^ All cases of young women (aged under 25 years), who died between 2014 and 2017 (the most recent closed case years at the time of data request, 2020) whose death was intentional self-harm (suicide) in Australia (*n* = 431), were reviewed. Young women were chosen due to their high rates of suicide and suicidality which include thoughts and plans of suicide and experiences of non-fatal suicide (AIHW, 2023b, 2023c, 2023d). The availability of such data means that they were most likely in contact with services prior to their deaths by suicide. These data were collected as part of a wider study about the role of gender in how communications of suicidalality among young women and girls were responded to, including what systemic issues in service provision exist for providing adequate supports for young women. Systemic issues appear in both the service context and social context in which communications of intent occur. These contexts are the frame for this study which focuses specifically on the role of gendered expectations among mothers who were suicidal and the role expectations of mothering may have played in how their suicidality was responded to. Data from 373 cases (87%) were available for analysis. A total of 58 had either no data beyond how the body was found, no digital files within the case, the person was not a woman, or the case was not a suicide. A total of 31 cases were noted to have mention of motherhood and were included in the qualitative analysis.

Each individual case in the NCIS may include up to four digital reports by people who were involved in the investigation of the suicide. These reports include a police narrative of circumstances which usually includes data from friends and family of the person who died; a forensic pathologist’s autopsy report which may include background data provided by police or medical records; a toxicology report which details any substances (medication, licit or illicit) found in the person's blood and/or urine; and the coroner's findings regarding the intentionality of the death (whether it was suicide or not), which may include extensive records if an inquest is held, including interviews with persons involved with the case interviewed for coronial purposes. Coronial cases can, therefore, provide abundant resources for qualitative analysis. The cases included in this analysis had anywhere between one and all four of these reports and varied greatly in detail.

It is not always clear in the NCIS whether or not someone was First Nations because there is no systematic collection of this information or data on other ethnicities on the NCIS. Though the NCIS does include a “country of birth” field, it does not indicate ethnicity. An “Indigenous origin” field is available from the NCIS only by special request and approvals. As First Nations identity was not a requisite for this paper, this field was not requested. However, some of the cases did mention that the woman was First Nations. This information would have been derived from friends and/or family. Australian statistical collections are usually reported as “First Nations” and “non-Indigenous” as part of the Close the Gap strategy (AIHW, 2023e).

A limitation of this study is that the data used are reconstructions of events and often based upon the perspectives of friends and family who knew the young women through a reporting of what may have occurred to people involved in the investigation, police, pathologists, and the coroner. Furthermore, this study uses a small number of cases which were analyzed qualitatively. It is therefore not generalizable to all deaths by suicide among young mothers but can provide important context for understanding why some young women died by suicide.

### Qualitative Analysis of Cases

All cases were reviewed and noted for mentions of children or pregnancy (motherhood). All names and identifying information have been changed or omitted. I included mothers whose children were in vitro, born, adopted, step-children, or children who had died.^3^ Documents from files were uploaded into NVivo 12 and coded. The initial codes were then reviewed, and overarching themes were identified. Rather than being a linear, step-by-step process, a frequent revisit and review of the cases, coding, and themes was conducted (Charmaz, 2014). Once a theme was identified, theories of motherhood were explored, and Hays’ (1996) theory of intensive motherhood was used to inform the analysis. These findings are presented below.

## Results

Findings have been organized into three key themes. The first theme, “Single mum on benefits”: Failing motherhood and mothering without a partner, illustrates how being a single mother affected suicidality. The second theme, “When he encouraged her to seek assistance, she refused”: Failing intensive motherhood and mental illness and suicidality, identifies how treatment-seeking for mental illness was incongruous with mothering young children, which added to the perception that the women were “risks” as mothers, and explores how the idea of motherhood as protective against suicide was invoked within the cases. The third theme, “Unborn report”: Risky (un)mothers and the loss of care of children, identifies the effect of becoming “unmothered” that is losing care of your child or children, and how becoming unmothered, including via the impact of custody battles, played a direct role in the suicide of some mothers.

### “Single Mum on Benefits”: Failing Intensive Motherhood and Mothering Without a Partner

The stigma of mothering without a partner appeared to play a part in some of the young mothers’ deaths by suicide. Fourteen cases mentioned an intimate partner relationship breakdown. These women either did not know who the father of their child was, did not disclose it to family and friends, or put it on the birth certificate, or decided or were forced to raise their children as single mothers. A common source of stress among single mothers was financial issues arising from difficulties working due to the logistics of parenting.

Police described First Nations woman, Celeste, as a “single mum on benefits”, and other cases of young mothers were also described by police as being on “benefits” invoking a sense of the “Welfare Queen.” According to police, Celeste had been pregnant twice and terminated the first. Both pregnancies occurred while Celeste was in her mid-teens. Police reported that

the deceased fell pregnant again and her mother stated that if she was willing to get

pregnant at a young age she is old enough to move out on her own.

Celeste moved in with a family member who supported her and her child until Celeste's death, yet Celeste's mother's response referred to in the police narrative reveals how the notion of maturity and responsibility is a critical expectation of intensive motherhood. Here, Celeste's mother withdrew her support of Celeste who had transgressed ideals of mature, responsible motherhood. During a bout of postnatal depression, Celeste was subsequently “advised” to give her child over to child services, which will be discussed in the third section. However, her case indicates how expectations of intensive motherhood, such as the ability to provide for and be solely responsible for the child, were hers alone.

Another young First Nations mother of two, Claudia, died by suicide while pregnant following an affair seen as scandalous by her community. Expectations of intensive motherhood played a direct role in her suicide – in particular the role of being a good, monogamous partner. The father of Claudia's unborn child was quick to not only publicly “out” the pregnancy (which she had not yet decided she would carry to term) but also defer blame to Claudia in a public forum, invoking both the sense that she was sexually promiscuous and he was not to blame for being seduced. These connotations reflect the idea that a bad mother indulges her sexuality rather than concentrating on her family (Feasey, 2017). The stigma of Claudia's sexual behavior was evident when her mother reported to the coroner that:

[…] her daughter was very conflicted about the pregnancy and was ashamed about her poor decision. […Following the public outing of the pregnancy] [Claudia] was subsequently sent a number of nasty messages […], including from [her ex-partner's] mother, which had a devastating effect on her mental health.

The father reported to the coroner that Claudia “was very conflicted about termination and was distressed about messages she had been receiving about the pregnancy.” The last time Claudia's mother spoke to her, she said that “[Claudia] had decided not to keep the baby as she felt it would be too difficult on her children. [Her mother] reassured her daughter that she would support her decision.”

Claudia was found dead by suicide by her two young children. Had Claudia not been publicly vilified by the father of her unborn child, she may have avoided the stigma and shame of the affair, as well as the association of that stigma onto her children. It would seem from the final discussion between Claudia and her mother, despite her own mother's support, that she already embodied the shame she wanted to spare her children. Her death may also indicate a potential extreme of intensive mothering, where the expectation for a mother to devote herself to her children went to the point of a self-sacrifice to break the shame of *her* “poor” decision. The biological father had absolved himself from blame and also been absolved of it by the discourse of intensive motherhood.

Other single mothers became single after the births of their children, a situation that often intensifies the need to work for money. One aspect of intensive mothering is the need to provide “the best” for children in all possible ways, which usually includes participation in paid work while also being expected to be the primary caregiver to the children (Hays, 1996). The logistics of working and mothering became acute and played a direct role in the suicide of Tracy who had recently become single. On the day Tracy died, she had been desperately trying to locate the father of her two-year-old so she could go to work. Her increasing frustration and desperation were noted in a text to a friend:

Omfg [oh my fucking god] why wont anyone answer me my bosses are really upset coz i cant go to work ahhhh fml [fuck my life] all i needed was for [ex] to be back for me to go to work fucking over this. no one gives a fuck if i lose my job hes declining all my calls now.

Tracy's exchange with this friend repeatedly mentioned she wanted to suicide.

These cases show how expectations of intensive mothering include a sense of responsibility towards (male) partners, non-promiscuity, sacrifice, and expectations of providing for the child both emotionally and financially. These women's issues were less with their children specifically and more with being able to provide an environment that meets the standards associated with intensive motherhood. Celeste was unable to stay at home with her own mother, who withdrew her support because Celeste was a teenager and pregnant. Claudia, shamed by her unborn child's father and her community, feared that shame would extend to her other children. Tracy did not want to continue into a future reliant upon a man who did not care about her or their child.

### “When He Encouraged Her to Seek Assistance, She Refused”: Failing Intensive Motherhood, Mental Illness and Suicidality

Experience of mental illness was prevalent among the young mothers in this study. Twenty-two of the young women's cases mentioned they were diagnosed with a mental illness, seven had been to hospital for mental illness or suicidality,^4^ and at least 10 mothers had previously tried to suicide.

Some informants in the coronial process described the young women as acting irrationally regarding their medical care. Following the suicide of young First Nations woman Patricia, a clinical review was undertaken by her local hospital and reported to the coroner after she had repeatedly been admitted for suspected domestic violence. They noted:

It is difficult to make adequate assessment given [Patricia]'s consistent denial of problematic alcohol use, as well as denial of family violence and mental health concerns. [Patricia] declined social work assistance when this was offered and had a history of poor attendance to appointments.

These statements place the responsibility upon Patricia to ensure that she and her children were in a safe environment, to the extent that her poor performance as a patient repeatedly precluded her from getting help for herself and her children. Jane, another young First Nations woman with two young children, was worried about seeking help. She had been put in foster care herself, during which time she was raped and threatened by the perpetrator. She was in a violent relationship and was reportedly apprehensive about seeking help of her suicidality out of fear for her children being removed. The coroner described:

According to [Her ex], [Jane] often self-harmed by cutting her arms, and had spoken of committing suicide, however, when he encouraged her to seek assistance she refused as she feared [child services] would find out and take her children.

This notion that the young mothers were supposed to disclose their suicidal thoughts to health practitioners (people with the power to remove their children) and the subsequent blaming of them for not doing so was also evident in other cases. In Chelsea's case, a young mother of four young children, the coroner noted that she had been to her local health clinic over 100 times in the years before her death presumably mostly due to her pregnancies and births as well as multiple reports to child services, which her GP apparently knew about. However, the coroner noted that she never brought up suicidality:

Her last appointment took place on [date]. [GP] reported awareness of [Chelsea]'s previous abusive personal relationships, and her difficulties with [child services] and her children. On [date], a Mental Health Care Plan [was developed] to cope with these difficulties, but [GP] noted that [Chelsea] never mentioned suicidal thoughts or plans.

Though many of the young mothers sought help, some received minimal help or did not continue with follow-up. Obtaining and enacting expert advice is an essential part of intensive motherhood (Hays, 1996), and though Hays explored this theme in regard to seeking parenting advice, it rings true here for mothers seeking medical help because doing so can sustain and maintain intensive motherhood.

#### “Her Children Were a Protective Factor for Her”: Failing Motherhood and Suicidal Crises

In contrast to situations where women avoided seeking medical attention or fully disclosing the extent of their anguish for fear of losing their children, 12 cases mentioned their intention to suicide before their deaths. Three of the cases mentioned missing their children as a reason for their intention to suicide while others were distressed about their loss of intimate relationships. Two young women, Claire and Valentina, explicitly used the notion of motherhood as a protective factor against suicide in different ways. However, in another case, the notion of intensive motherhood as protective against suicide was also invoked, albeit less explicitly, by the person the young woman turned to for help. As discussed above, Tracy discussed her wish to suicide via text messages to a friend.

In the excerpt below, Tracy's friend asked her to put her child's need for her to live beyond her desire to die and tried to reinforce a sense that she, as a good mother, would do that. The police recorded her last received phone messages:

[Friend]: No one else deserves your life. Except for your [child] […] [who] deserves to have [their] mother taking care of [them] and loving [them]. […] If you can’t stay for me, stay for [child's name]. That little [kid] needs [their] mummy […] [Tracy]?

The friend references expectations of intensive motherhood by saying that it is Tracy's child who “deserves her life” (not Tracy herself) and for her to be taking care of the child. The underlying expectation here is that Tracy owes it to her child to be the kind of mother her child needs and that she should live for her child’s sake. The same friend subsequently went to her house and found her dead and her child asleep.

Tracy's other texts indicated her deep frustration with the father of her child, how she felt humiliated that he rejected her and feared that she would never be loved again. This, and her family and friend's insistence to stay alive for her children, invoked the extent to which the “good family” is associated with good motherhood. This marginalizes discussion of what, for Tracy, her real issues were: the loss of her partnership and the realities of single motherhood with an unsupportive co-parent.

Claire was a single mother of three young children. She attended her local emergency department for suicidal ideation. The emergency department triage nurse recorded that

[She] was feeling “down and out.” She was noted to be teary and not coping with her children. [The doctor who saw her noted that:] [Claire] appeared to be a very loving and capable mother however she was worried that she was not doing enough for her children and that if her depression continued to worsen she would be unable to care for them.

As the triage nurse's assessment demonstrates, Claire, who was seeking help for suicidality, was described as a “loving and capable mother,” showing that her attempt to get help reflected on her status as a good mother. This was also used as a means to undermine what she was seeking treatment for: suicidality due to the need to care for her children*.* The following is from a doctor who saw Claire after the triage nurse on the same night she sought help at the emergency department:

[Doctor] considered [Claire]'s appearance and behaviour was appropriate, although she was tired she was alert and responsive. Her mood was clearly depressed, and her affect was congruent with her mood. […] [Doctor] considered her insight and judgment to be intact and appropriate, as she was seeking help and was aware of her mental illness. […]. Her protective factors included her sense of responsibility to her family, love for her children and appropriate help seeking.

It ought to be noted that it does not appear, from the assessment, that Claire considered her children to be protective, but that the doctor had concluded this from their observations and likely because of their expectations of “good” mothers. Two days after Claire was discharged, she went to a different hospital for suicidality:

[Claire] requested a mental health review as she was feeling overwhelmed at home caring for her children and household duties. [… She] stated that she felt unbalanced, confused and exhausted. Her presenting complaint was documented as suicidal ideation. […] After review, [Claire] was discharged home with her mother at [time] and was advised to contact mental health services if she required.

Claire died by suicide the next day. It does not seem surprising that Claire did not seek crisis help for a third time, given that she had tried twice to get help and was told she could “contact mental health services if she required.” Both times, hospital staff dismissed her concerns on the basis that motherhood is protective and that she would continue to be a “good” mother and therefore a good patient. Following her suicide, the hospital was asked to provide a statement to the coroner which outlined the reasons they did not admit her for inpatient care. The chief medical officer reported that

[Claire] was not deemed to have immediate further risks of self-harm or suicide because of the absence of any prior suicide attempts, the lack of a current plan, she was not displaying current signs or symptoms of a major mental illness, having protective factors of three young children, she strongly denied plan or intent during assessment and was discharged to the care of family, who were willing to support her.

Claire's file indicated a fatal disconnect: she repeatedly stated that she was struggling with her children, yet services insisted that motherhood was a protective factor against her suicidality. Her case illustrates how notions of motherly love can be a reason a woman might believe she needs to die by suicide, as she sees herself as inadequate, unable to cope, and unable to get help (Biggs et al., 2023). Claire repeatedly reported that she was concerned she could not care for her children any longer, yet the fact that *she had to care for them* was deemed one reason it was presumed she would not suicide.

In contrast, Valentina, a young mother of two primary school-aged children, invoked the “protective” factor of her children in order to be discharged from the hospital following a non-fatal suicide. She reportedly told the psychiatrist that

She was remorseful for her actions and indicated her children were a protective factor for her. She agreed to mental health community involvement and denied any further thoughts of suicidal ideation or self-harm.

Valentina died by suicide that evening while her family slept.

Tracy's story highlighted failed attempts from a friend to appeal to normative expectations that a mother would never leave or abandon her children, and her friend assumed the idea that motherhood would be a factor preventing Tracy from suicide. Claire and Valentina's situations invoked the notion of intensive motherhood as protective in different ways. Claire was attempting to meet the standards of intensive motherhood by seeking help, and indeed was seeking assistance because she was concerned she was failing to meet the standards of care expected of her. Yet this help-seeking was used as evidence of her good mothering, and her children as protective. Valentina, instead, used the discourse of intensive motherhood being protective of suicide as a means to suicide. Valentina's two children were school-aged and therefore well outside the perinatal period.

### “Unborn Report”: Risky (Un)Mothers and the Loss of Care of Children

Becoming unmothered by the removal of children by state authorities was another issue that emerged from the analysis of these cases. The removal of one's children by the state is one of the starkest instances of failed intensive motherhood, as the woman herself is deemed a risk to her children by being deemed incapable of protecting or caring for them (Gross et al., 2014). The loss of care of children was evident for women deemed to be “risky,” common among young women who were in violent relationships, as well as those using illicit substances before, during, and/or after pregnancy. When some women could not provide a safe home for their children or if they maintained their substance use, they transgressed expectations of intensive motherhood in fundamental ways, so fundamental that it attracted the legal power of the state to remove the children. This section explores the role this transgression played among young women who died by suicide, firstly, through the effects of the loss of care of a child to a violent partner, and secondly, how custody issues led these women to experience suicidality. In both situations, the penalty was borne by the women.

#### “The Tipping Point”: The Role of Becoming Unmothered by Violent Partners

Children were often removed from the young woman's care due to alcohol and substance abuse by the young woman and/or her partner, or due to violence within the household. Alcohol and/or illicit substance abuse were mentioned in 10 of the cases. Victimization was common among the cases—19 files mentioned the women had been victimized (including physical and mental violence, bullying, theft, or vandalism). Of these 19 cases, 17 mentioned they had been the victim of physical violence, including sexual assaults, usually perpetrated by a partner. For these women, their children being taken away was noted by friends and family to be a “tipping point” in their distress. In some cases, the women blamed themselves for circumstances beyond their control.

The threat of or having been “unmothered” due to the actions of their partners played a direct role in some young mothers’ suicides. This could be due to the stigma of failing to live up to intensive motherhood expectations of protecting their children above all else because their children were injured by other people the women allowed into their lives. This immense distress was clearly evident for Mimi, who died from suicide the night she was told her child would be permanently removed from her care because of her abusive partner. Her suicide occurred following rather paradoxical circumstances. Following an assault by her partner on Mimi's child, which resulted in police being called to their home, the coroner explained:

While the deceased's partner was being assessed the deceased attended [local] Police Station to provide a statement in relation to incidents of physical abuse of her [child] by her partner. The deceased's partner was subsequently charged in relation to the assaults. [5 days later …] the deceased called a detective at [local police] office to confirm she may be charged in relation to her failure to prevent or report the assaults against her [child]. The detective advised the deceased the investigation was ongoing, but it was possible she may be charged.

Mimi was advised later that same day that a permanent court order was in place preventing her from access to her child. She died by suicide that evening. Mimi's violation of the expectation that she would prevent violence to the child, which implies a certain amount of power and responsibility which may be unlikely to be present in a violent relationship, had ended in the removal of her child from her care and the threat of prosecution for not reporting the violence—despite being the person who brought it to police attention in the first place.

In similar circumstances, Tess felt she needed to leave her toddler with her violent ex-partner, who was the father of her child. She did this so as not to disrupt their child's routine and because she had nowhere permanent to live. Her decision indicates a level of intensive motherhood: she put the child's assumed need for stability ahead of her own wellbeing. The father then refused to allow her to see their child and made an almost daily point of taunting her about it for months until her suicide. He took all the money in their shared accounts, meaning she was unable to replace her broken-down car. This meant she was unable to get to work, unable to find affordable accommodation, and unable to see her child. Her ex-partner effectively made her unable to mother, which grew increasingly unbearable for her. She missed Christmas with her child as well as the first day of school “because she did not have access to a car, and because the intervention order was still in force, meaning that she could not be near [Ex-partner].” After five months of not being able to see her child, her choice to suicide was directly related to her role as a (un)mother. The coroner noted that

The date of the Deceased […] suicide was her [child name]'s birthday. The father [Ex-boyfriend] refused the Deceased access to see the child for [their] birthday. It is believed that this may have been a tipping point causing the Deceased to take her life.

While these women lost the care of their children due to their partner's violence and abuse, some of the other young mothers were unmothered due to their own actions, explored in the next section.

#### “I Can't Keep Saying Goodbye”: Risky (Un)Mothers and Loss of Custody

As noted above, Celeste was a young First Nations single mother. The police narrative linked to Celeste's suicide highlights the colonialist and racist attitudes still prevalent toward First Nations mothers and First Nations people generally. After the birth of her child while she was a teenager, Celeste was diagnosed with postnatal depression. She sought help for this, and police noted that “on the advice of a health worker, she placed her [child] into voluntary care.” Celeste's First Nations status and age almost certainly played a part in her being advised to have her child placed in care by a health professional. She had violated multiple expectations of intensive motherhood: she was not white and middle-class, she was a single mother, and she was a teenager. All of these create dimensions and reasons for her to be deemed an irresponsible, unworthy mother, and her postnatal depression was analyzed within this frame. At this point, she was living with an extremely supportive family member and seemingly doing well. After her child was placed in care, however, the police wrote:

The accused^5^ continued to have meetings with [child services] and Health workers to ensure she was suitable to raise her child. As a result, the child was returned home but the NOK [next of kin] was named as the carer. This incident caused the deceased to become more stressed which lead [sic] to another self harming incident by way of cutting and began to drink heavily and smoke cannabis on the daily.

The investigation into Celeste's death documented a spiral of her behaviors. After losing custody of her child and regaining it, she remained unmothered because she was not officially labeled the “carer.” Despite assessments regarding the suitability of returning the child home, Celeste's status as a mother was denied. Celeste was constrained by contradictory expectations of motherhood, to put her child above all else and therefore give up care, but she was then recognized by health services as no longer a mother. These contradictions, experienced through the expectation that Celeste prove that she was a suitable carer and mother, affected her profoundly. Following her loss of status as mother, Celeste began self-harming and engaging in drugs and alcohol. She died by suicide some years later.

Joan typified the “risky” mother. She met the father of her child in a substance rehabilitation facility and they “were known to frequently use drugs together.” During her pregnancy, her child was flagged in an “unborn report” which detailed how Joan had already failed the expectations of intensive motherhood before the birth of her child. In addition to her drug and alcohol use, concerns were raised regarding her “transient lifestyle” with her partner while she was pregnant. Joan violated expectations that pregnant mothers do not use dangerous or illicit substances and that a mother should provide a stable home in which she can devote herself to her child. But Joan herself was the one who revealed this to health workers when she admitted to drug use while asking for help. This indicates some levels of attempting “good” motherhood as she was seeking help from an appropriate source and disclosing her issues.

Joan was involuntarily admitted as a mental health inpatient while she was in labor because she would not allow staff to intervene when her baby grew distressed during birth. Joan was thus “assessed as being at risk”, yet it was not mentioned in her coronial file what she was deemed to be “at risk” of. It is not clear from Joan's case notes whether she was suffering a mental illness when she gave birth as there was none mentioned. Her child was forcibly removed from her care through a court order by hospital staff two days after birth and placed into the care of her parents. Having just given birth and being held involuntarily at the hospital, Joan was unable to contest the order in court. Over the next few years, Joan managed to become drug-free and lived with her parents and her child. However, she was repeatedly prohibited from full-time custody, sometimes for reasons beyond her control. On the night Joan died, she was unable to stay at her parent's home with her child after some violent incidents between her and her sister. The coroner noted that Joan

had asked to conduct a video call with [child], so [her mother] and [her sister] called her so she could see [child]. […] [Joan] told [her mother], “Mum, I just can’t do this anymore. I can’t keep saying goodbye to my [child]. I've had enough.” She then ended the call.

Joan's last words to her mother resonated her hopeless situation. Following the call, her mother and sister grew concerned, went to her house, and found her dead by suicide.

## Discussion and Conclusion

Using Hays’ (1996) theory of intensive motherhood, this paper explored expectations of ideal motherhood to broaden our understanding of young mothers who died by suicide, despite the assumption in research and practice that motherhood acts as a protective factor against suicide. Furthermore, despite the focus on the perinatal period as a risk factor for suicide, an important finding was that most of the young mothers who died by suicide did so outside of the perinatal period. Significantly, through the use of qualitative analysis techniques, which remain the exception in suicide research, the paper has shown that expectations of intensive motherhood can have direct influences on why some women died by suicide. This paper also broadened understandings of suicide among young women through an analysis of gendered expectations. Overall, this paper has added to two important areas of suicide prevention research: suicide among young women and maternal deaths by suicide.

Some limitations of this paper were that first we are unable to know whether what was reconstructed during the coronial process is what occurred and second we do not know whether the circumstances described by informants are how the young women themselves would have perceived them (Canetto, 1997; Scraton, 1999). These reconstructions can also be influenced by sexist, racist, and classist assumptions about the women by the people who knew them and those who investigated their deaths (Scraton & Chadwick, 1986). The cases included details thought pertinent to help investigate intentionality of death by those who reported the suicides. Therefore, the women's experiences are reconstructed retrospectively multiple times within the cases by those involved in the investigation *after* they had already died by suicide with the goal of determining *whether* they died by suicide. Furthermore, the exploration of race and ethnicity was limited to First Nations status only; as other ethnicities are not routinely collected in the NCIS. Exploration of the role of ethnicity, intensive motherhood, and suicidality needs further research.

This paper demonstrates how expectations of intensive motherhood can have fatal consequences. It highlights the need for suicide prevention efforts among mothers to focus on the social needs of women. A common issue among young mothers who died by suicide was domestic violence and the loss of custody of children (or the threat of losing children), as well as relationship breakdowns, which played a major role in how some of these women perceived themselves as mothers, the types of services they accessed for help, and the way that they received treatment and care.

An expectation of intensive motherhood is that a mother should do anything for her child. If a mother is unable to do so, for example, due to domestic and family violence or drug and alcohol issues, mothers can be seen as at risk and their status as mothers may be revoked through social and legal sanction, or via one of the most significant sanctions the state can use against its citizens apart from incarceration—the removal of one's child. The author is not questioning the decisions of child protection authorities in these cases, but rather highlighting that these situations demonstrate a link between norms of motherhood, shame, and suicide, which are more complex than the focus on mental illness—the dominant trope in responses to suicidality. One could add, however, that casework involving women who are drug and alcohol dependent and mothers is a complex area of practice. It is too simple to expect that the power of substance dependence should vanish the moment a woman finds out she is pregnant and her love for her child should overpower that dependence. This gives little consideration for why substance use might be an issue for these young women in the first place, which may be due to a myriad of adversities and related stigma (Guinle & Sinha, 2020; Meyers et al., 2021). In turn, this suggests that addressing issues around access to support and exposure to violence, which are clearly evident for these women certainly, and supporting them in their mothering role by dedicating the time, compassion, and empathy required to support them to get sober, may need to be addressed in lock-step with protecting the child. The expectation that young mothers seek treatment for these conditions is inherently challenging because their children depend upon them, which can increase stress and in turn exacerbate alcohol and substance abuse and/or dependence, as explored in the results.

A key aspect of Hays’ (1996) theory of intensive motherhood is the expectation that a *mother* provides a safe environment for *her* children. The criteria for safety can be imbued with sexist and racist undertones, as seen in Celeste's case in which she was advised to give up care of her child because she was experiencing postnatal depression. The fact that she was young, single, and Blak^6^ almost certainly played a role in this advice. The rate of First Nations children being removed from their homes in Australia has increased in the last decade and is now ten times higher than the rate for non-Indigenous children (Liddle & Gray, 2021). Celeste's story indicates contradictions in expectations of intensive motherhood by showing that she was both listening to and following advice for her child, yet unable to renegotiate this advice. Her story also shows the powerlessness of many young women within the human service system, particularly when they are mentally unwell. It also shows the application of human service providers’ coercive power over some women's decision-making (Townsend, 2023).

The responsibility of providing a safe environment for children was also made acute among young mothers who experienced domestic and family violence. Mothers are expected to be the person who stops family violence, the person expected to seek refuge, the person expected to protect “her” children (Singh, 2021), while the perpetrator of the violence, the person creating an unsafe environment, is allowed to continue to do so *because* the mother remains there with the child/ren (Bordo, 1993; Cramp & Zufferey, 2020). Patricia's story showed that doctors at her local hospital suspected she was experiencing family violence, yet she was ultimately painted as irrational and responsible for the doctors’ inability to help her. Her story crystalized the neo-liberal assumption that Patricia was acting entirely under her own free will, obscuring the cultural and organizational systems which sometimes apply power in gendered and racialized ways (Bugden et al., 2021). We saw that some women who did ask for help in these situations were sometimes blamed by authorities and criminalized themselves, as in the cases of Mimi and Celeste, although Celeste was not a criminal, she was described by the police as “the accused.”

Medical treatment can be coercive (Townsend, 2023). Some mothers, like Jane, genuinely feared their children being removed from their care—a fear which is quite rational, especially among Aboriginal and Torres Strait Islander people as this practice continues today (Liddle & Gray, 2021). Patricia, a First Nations woman who was dealing with substance abuse issues and experiencing intimate partner violence, and Chelsea, a survivor of abuse who was having trouble with child services, were both under immense pressure but never mentioned suicidality to health professionals. The shame around being a mother contemplating suicide and suffering significant mental distress can create a silence among women, even when in frequent contact with health services (Biggs et al., 2023). Rather than motherhood being a source of assistance, fear of what consequences might result from disclosing their concerns may have caused these women to hide their distress. That is, their silence may have been *because* they were in frequent contact with health services. This shame may stem from expectations regarding appropriate motherhood, captured in discourses of intensive motherhood. The reality that a woman may reject this role—preferring death—breaches fundamental social norms and expectations associated with good mothering. As a powerful social force generating shame, these experiences may exacerbate feelings of suicidality (Biggs et al., 2023).

The contradictory expectations of intensive motherhood, to look after children through self-sacrifice while also maintaining medically sanctioned self-care, in order to attempt to live up to norms associated with intensive motherhood, may have created tension and silence among young women and the health professionals they turned to for help. Health professionals may not have raised the issue of suicidality due to the assumption that motherhood is protective against suicide, despite these women experiencing multiple risk factors for suicide: including experiencing intimate partner violence, family violence, drug and alcohol use, and contact with the justice system (AIHW, 2023f).

The notion of motherhood as a protective factor was integral to the care of two young mothers, Claire and Valentina, who presented to the hospital for suicidality hours before their deaths by suicide. There was an implication that intensive motherhood was something the women *wanted* to do. Claire presented to the hospital for suicidality because she was unable to cope with the care of her three young children. Despite demonstrating that the caregiving role was causing her distress, the staff assumed that her role as a mother was a protective factor against her suicidality. This assumption, that mothering is protective, transferred from a cultural norm and stereotype into criteria for clinical assessment, despite there being little and contested evidence that mothering is protective among women experiencing mental distress. This is important because suicide risk assessments include reference to parenting and the perinatal period as protective factors against suicide (see, for example, section 2.2.2 “protective factors” of Department of Health, 2010:18). While the parenting role is relevant, whether it is protective, a cause of distress, or something else, must be carefully determined on a case-by-case basis. Assessment should not be based on assumptions of gender and motherhood, particularly when there is little to support this supposition during the perinatal period (Al-Halabí et al., 2021) and no evidence to support it after the perinatal period.

Further, several women who were not coping with the expectations and realities of motherhood, particularly single mothers, were oftentimes diagnosed as mentally ill. This conflation of a failure to cope with expectations of motherhood as a form of mental illness has been historically used against women, especially working-class women, and women of color as a source of shame (Appignanesi, 2008; Fattore & Mason, 2020; Mason & Fattore, 2017). Once diagnosed, expectations of treatment for mental illnesses were incongruous with the mothering of young children for these women. While this may not be the situation in all forms of mental health intervention, this construction of mental health treatment as incompatible with mothering certainly was a factor in the suicide of several women because it placed the women in a bind: in order to be better mothers, they needed to be better patients, but in order to be better patients they needed to put their needs before their children's, which would transgress “good mothering” and therefore be read as further evidence of the mother's mental ill health.

Moreover, this bind also prevented some women from seeking help because they feared that their position would be made more precarious if they admitted the extent of their distress to the service system. Jane's case exemplified the fear young mothers may have had around telling health professionals of their mental distress, which could heighten the risk of having their children removed from their care, with this fear becoming a reality for Celeste and Joan. This echoes findings by Busch and Redlich (2007), which indicated some mothers in treatment were worried that if they were unable to adhere to their treatment, this too would be reason enough to remove their children from their care. Similarly, some of the women whose cases were assessed in this paper were reticent to seek help for mental health issues for fear of losing their children and the stigma of needing mental health treatment in relation to motherhood (Busch & Redlich, 2007; Moore et al., 2016). These findings confirm the need for trauma-informed, anti-sexist, anti-racist, and social issue-focused treatment approaches which do not blame women for their circumstances. It is through these approaches that women can be supported in their mothering role and their children's safety guarded and stigma reduced. Such approaches may result in fewer young women taking their own lives because of the stigma in treatment-seeking and feeling they are failed mothers (Fook, 2012; Huo et al., 2023; Levenson, 2017).

## Notes

1. ‘Nonfatal suicide’ is used throughout this paper in lieu of the more common ‘suicide attempt’ due to the implication that an attempt is less serious, incomplete, and unsuccessful. (Canetto & Lester, 1998; Canetto & Sakinofsky, 1998)

2. Ethics approval was sought and approved by the [author’s] University’s Human Research Ethics Committee (HREC) Humanities and Social Sciences (HASS), reference number 5201834315018; The Justice Human Research Ethics Committee (JHREC), reference number CF/18/30054; and the National Coronial Information System (NCIS), reference number M0425.

3. All children are referred to in gender-neutral terms (e.g., they, them, baby, toddler, and child) and their ages are omitted.

4. The intention to suicide is not always clear and may be referred to as the more generic ‘self-harm,’ which does not always include the intention to die.

5. Celeste is referred to as “the accused” several times throughout the police narrative in lieu of “the deceased” or her name. I read this misidentification as an indication that the police officer writing the report held deeply racist views towards her.

6. ‘Blak’ is a term for First Nations Australians to differentiate their experiences from those of other communities of colour (see Latimore, 2021).
